# Outcomes of Surgery with Vaginal Native Tissue for Posterior Vaginal Wall Prolapse Using a Special Technique

**DOI:** 10.25122/jml-2020-0093

**Published:** 2020

**Authors:** Samira Sohbati, Maryam Hajhashemi, Tahereh Eftekhar, Maryam Deldar, Nahid Radnia, Zinat Ghanbari

**Affiliations:** 1. Department of Obstetrics and Gynecology, Faculty of Medicine, Kerman University of Medical Sciences, Kerman, Iran; 2. Department of Obstetrics and Gynecology, Faculty of Medicine, Isfahan University of Medical Sciences, Isfahan, Iran; 3. Department of Obstetrics and Gynecology, Imam Khomeini Hospital, Tehran University of Medical Sciences, Tehran, Iran; 4. Department of Obstetrics and Gynecology, Faculty of Medicine, Hamadan University of Medical Sciences, Hamadan, Iran

**Keywords:** Pelvic organ prolapse, rectocele, enterocele, prolapse surgery

## Abstract

There are several techniques for repairing prolapse in the posterior vaginal compartment, yet there is no general agreement on the best surgical procedure. This study was performed to investigate the outcomes of the common vaginal route technique for posterior vaginal wall prolapse repair in the first Iranian fellowship teaching center for female pelvic floor disorders. This prospective cohort study was performed on women with posterior vaginal wall prolapse with or without prolapse of other vaginal compartments who underwent surgery between 2014 and 2018 in a referral center for female pelvic floor disorders. A follow-up period of 12 months was considered. Patients subjected to the transvaginal technique by attachment of the rectovaginal fascia to the pericervical ring using vaginal native tissue were included. Among the 107 patients, the Pelvic Floor Distress Inventory-20 (PFDI-20) scores were 141.87 ± 34.48 and 100.87 ± 26.48 before and after surgery, respectively, showing the significant improvement of patient’s symptoms after surgery in the 12-month follow-up. Comparing Pelvic Organ Prolapse Quantification (POP-Q) results before and after surgery, a significant improvement in patients’ conditions was seen at the 12-month follow-up. Based on the results of the present study, the surgical procedure of the rectovaginal fascia attachment to the pericervical ring in posterior vaginal wall prolapse repair seems an effective surgical intervention without significant morbidity in the short-term follow-up.

## Introduction

Pelvic Organ Prolapse (POP) is one of the most common gynecological dysfunctions that adversely affect women’s quality of life by limiting their physical and psychosocial activities and sexual function [1].

According to the International Urogynecology Association (IUGA) and the International Continence Society (ICS), prolapse refers to a falling, slipping, or downward displacement of a part of an organ. Pelvic organ refers most commonly to the uterus and/or other different vaginal compartments and neighboring organs such as the bladder, rectum, or bowel [2].

With respect to the occurrence site, POP may involve the anterior vaginal wall, posterior vaginal wall, and vaginal apex (apical prolapses). Also, POP may occur in one or more compartments [3].

Commonly, the posterior vaginal wall prolapse refers to rectal protrusion into the vagina (rectocele). Higher stage posterior vaginal wall prolapse after prior hysterectomy will generally involve some vaginal vault descent and possible enterocele formation. Enterocele formation can also occur in the presence of an intact uterus [2].

There are variations in the prolapse incident and prevalence of each of the vaginal compartments as different studies are using different methods and populations (symptomatic or asymptomatic).

Patients with posterior vaginal wall prolapse with or without prolapse of other compartments may potentially develop posterior enterocele, rectocele, or sigmoidocele [4].

Some patients with rectocele may be asymptomatic, while others may show symptoms such as pelvic pain/pressure, posterior vaginal bulge, obstructive defecation, incomplete defecation, constipation, dyspareunia, or erosions and bleeding of the mucosa if there is tissue exposure to the outside environment [5]. The characteristic symptoms of enterocele are emptying difficulty, post-evacuation discomfort, and pelvic pain or heaviness [6].

Variable POP degrees have been indicated with the clinical examination of 41% to 50% women, although only 3% of these patients reported their symptoms [3].

In a study on 3730 Iranian women aged 16 to 68 years, the overall prevalence of POP was reported 53%, most of which were stage 1 or 2 of the disease based on the Pelvic Organ Prolapse Quantification System (POP-Q)[7].

The actual incident rate of rectocele is unknown, but asymptotic posterior vaginal compartment prolapse has been reported in about 40% of parous women [8].

The exact incidence of enterocele is also unclear, although its incident rate varies between 11% and 45% in patients with pelvic floor dysfunctions [9].

The posterior vaginal compartment is the site that is frequently operated on, and its surgery is often accompanied by the surgery of other vaginal compartments [10]. In literature, various methods for the rectocele and enterocele surgical treatment have been described as transvaginal surgery with or without using graft or mesh, transabdominal, or transanal surgery.

On the other hand, the effects of the simultaneous presence of rectocele and enterocele on surgical treatment outcomes of these defects are not clear [4].

Imam Khomeini Hospital Complex is the teaching hospital of Tehran University of Medical Sciences, and its center for female pelvic floor disorders (PFD) has been the founder of fellowship training for female PFD in Iran since 2012. This department is the tertiary referral center for women with PFD. At this center, rectocele and enterocele surgical interventions via the vaginal route are performed using rectovaginal fascia repair and its caudal attachment to the precervical ring.

In our research, we described objective and subjective surgical outcomes of posterior vaginal wall repair using the common vaginal method in the center that this study was performed.

## Material and Methods

This prospective cohort study was performed on women with rectocele and/or enterocele defects who underwent surgery between 2014 and 2018 at the Imam Khomeini Hospital Complex, one of the academic centers of Tehran University of Medical Sciences in Iran. This study was approved by the Ethics Committee of the Imam Khomeini Hospital Complex.

Inclusion criteria for this study were women with PFD symptoms whom POP-Q test revealed they had enterocele and/or rectocele with or without prolapse of anterior and/or apical vaginal compartments. These patients had no tendency for conservative interventions, or conservative treatments had failed in their case. Patients included in this study were followed up for 12 months after the surgery.

Obesity, history of genital or abdominal cancers, neurological diseases such as multiple sclerosis, and pelvic radiation were considered as exclusion criteria. Also, subjects who did not complete the follow-up were excluded as well.

Patients’ medical history was obtained using a form by a third- or fourth-year resident of obstetrics and gynecology accompanied by a fellow assistant of female PFD attending the PFD clinic of the Imam Khomeini Hospital Complex. These forms included complete demographic information, medical history, and history of PFD, including any problems of the urinary, gastrointestinal and genital systems as well as any POP symptoms based on the IUGA and ICS definitions [2].

In order to assess the PFD symptoms related to the quality of life, standard questionnaires such as the Iranian version of the Pelvic Floor Distress Inventory-20 (PFDI-20) were used. In this study, the Iranian version of PFDI-20 was used before and after the surgical treatment [11]. In this center, the PFDI-20 questionnaire is always completed by someone other than the patient’s surgeon.

To evaluate the prolapse severity, each patient underwent a POP-Q test and other comprehensive physical examinations.

In this center, the follow-up of patients undergoing surgical intervention for POP was performed 2 weeks, 2 months, and 6 months after the intervention, and then annually. A one-year follow-up was considered.

Patients’ follow-up information was also recorded at the Imam Khomeini Hospital Complex using PFD clinical forms (including postoperative medical history, physical examination based on the POP-Q system, and PFDI-20 questionnaire).

### Surgical technique

Patients underwent regional or general anesthesia. Half an hour before surgery, 2 grams of Cefazolin was injected intravenously as antibiotic prophylaxis. Then, the patients were placed in the lithotomy position. The vaginal examination was done under anesthesia. The same surgical technique was performed for all subjects under the supervision of a female PFD specialist surgeon.

In the beginning, a transvaginal hysterectomy (TVH) was performed if needed. Then, for the repair of posterior vaginal wall defects, this compartment was opened, and the rectovaginal fascia was separated from the vaginal epithelium. The anterior vaginal wall was then repaired as needed, and finally, the posterior vaginal wall and perineal body were repaired as well. Perineorrhaphy was performed in all cases. In the case of stress urinary incontinence (SUI), based on the urodynamic study and the patient’s informed consent, the transobturator tape (TOT) or tension-free vaginal tape (TVT) procedures were performed using a separate incision after perineorrhaphy.

If an apical suspension was required, either the high uterosacral vaginal vault suspension or sacrospinous suspension (using the Capio SLIM™ suture capturing device, Boston Scientific, MA, USA) were performed.

In order to repair the posterior vaginal compartment, a midline vaginal incision was performed from the lower third of the posterior vaginal wall to the posterior fourchette. Normal saline was then injected between the rectovaginal fascia and the vaginal epithelium.

Using the sharp dissection method, the vaginal epithelium was separated from the rectovaginal fascia at the entire surface of the posterior vaginal wall. The extent of separation was as far as the palpation of the cervix, below the posterior vaginal epithelium in the midline, and the palpation of both sides of the uterosacral ligaments laterally, as well as the complete separation of the rectum. In the presence of an enterocele, its sac was separated from the posterior vaginal epithelium. If the sacrospinous suspension was required, the right lateral dissection was performed as far as the right ischial spine.

**Figure 1:**
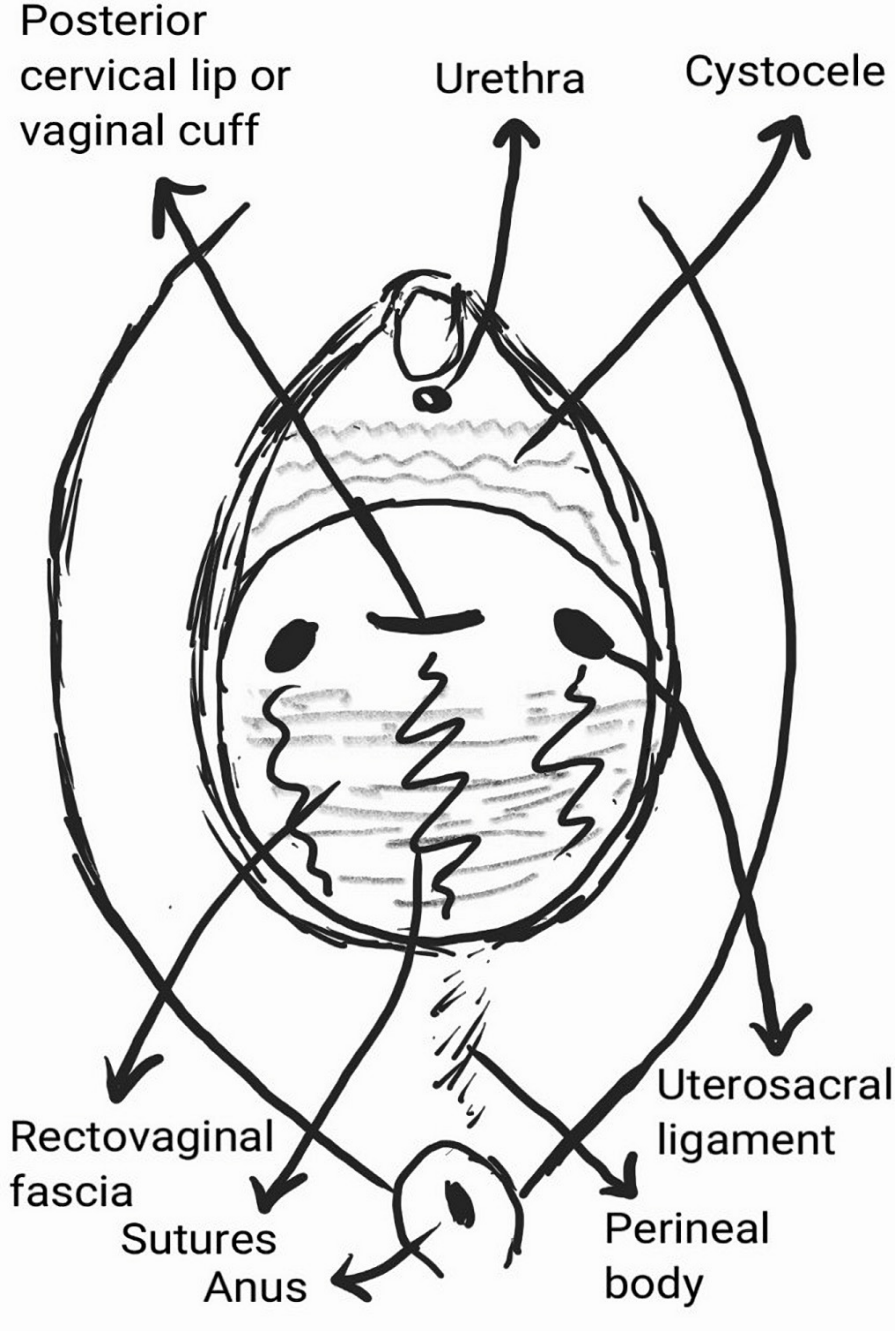
Diagram of sutures from rectovaginal fascia to the pericervical ring.

Then, rectovaginal fascial defects, in case of large transverse defects, were repaired using Vicryl 1 sutures. Afterward, for all cases, the rectovaginal fascia was sutured using the caudal pattern (from DeLancey level 3 to level 1) and stitched to the posterior cervical lip or posterior part of the vaginal cuff in the midline, parallel to the junction of the uterosacral ligaments. From both sides, it was sutured to the uterosacral ligaments using Polydioxanone 1. In this way, the rectocele and enterocele (if present) were repaired (Figure 1).

Before and after suturing, a rectal examination was performed in order to ensure that the rectal mucosa was not damaged or sutures were not inserted into it.

Then, the edges of the vaginal epithelium were smoothed, but excess vaginal mucosa was not excised. Finally, a running suture with Vicryl 2/0 was used for closing the vaginal epithelium and completing the perineorrhaphy. Levatorplasty was not performed in this method.

After finishing the complete repair, cystoscopy was performed to ensure that ureters were open.

Twenty-four hours after surgery, the urinary catheter and vaginal pack were removed, and after removing the urinary catheter, post voiding residue was measured for all patients under study.

### Statistical analysis of data

The data were analyzed using the IBM SPSS software (version 23). The mean PFDI-20 scores and its questions were examined using the paired-samples T-test. The relationship between POP-Q stages before and after surgery was assessed using the McNemar-Bowker Test. A p-value of <0.05 was considered significant.

## Results

During this study, 460 patients with PFD were accepted as suitable candidates for vaginal surgery. One hundred eighty patients with posterior vaginal wall prolapse with or without other compartment prolepses underwent vaginal native tissue surgery. However, follow-up for at least 12 months was possible for only 107 patients. The median age of subjects was 50 years (24-68 years; relative mean: 49.69 ± 10.95). Demographic data are provided in Table 1.

**Table 1:** Demographic characteristic of the study population, N=107.

Patient characteristics	Frequency (%)
Parity^a^	
**1**	3 (2.80)
**2**	22 (20.56)
**3**	17 (15.88)
**4**	25 (23.36)
**≥5**	40 (37.38)
**Menopause**	52 (48.59)
**Previous hysterectomy (abdominal)**	1 (0.93)
**Delivery method**	
**Vaginal**	96 (89.71)
**Vaginal delivery + cesarean**	11 (10.28)

^a^median= 4 (1-10).

Among the 107 patients, 15 (14.01%) patients reported large perineal laceration at the time of delivery but determining the severity of their condition was impossible because their unit summary sheet was not available. The vacuum had been used for 3 (2.80%) patients included in our study.

Table 2 shows patients’ symptoms before surgery and after the 12-month follow-up. The severity of prolapse based on the POP-Q score before and after surgery is provided in Table 3, and the frequency of the procedures is shown in Table 4.

**Table 2:** Pre-operative and postoperative pelvic floor symptoms (N=107).

Symptom	Pre-operative frequency (%)	Postoperative frequency (%)	Preoperative score ^a^	Postoperative score ^a^	P-value ^b^
**Bulging**	66.00 (61.68)	5.00 (4.67)	2.76 ± 1.44	1.11 ± 0.53	<0.001
**Splinting**	28.00 (26.16)	16.00 (14.95)	1.69 ± 1.23	1.34 ± 0.87	0.009
**Heaviness in pelvic area**	41.00 (38.31)	19.00 (17.75)	2.07 ± 1.41	1.40 ± 0.95	<0.001
**Obstructed defecation**	56.00 (52.33)	26.00 (24.29)	2.50 ± 1.48	1.60 ± 1.09	<0.001
**Incomplete bowel emptying**	55.00 (51.40)	28.00 (26.16)	2.47 ± 1.48	1.60 ± 1.09	<0.001
**Urinary frequency**	37.00 (34.57)	14.00 (13.08)	1.96 ± 1.38	1.28 ± 0.77	<0.001
**Urge urinary incontinency**	79.00 (73.83)	37.00 (34.57)	3.07 ± 1.33	1.84 ± 1.25	<0.001
**Stress urinary incontinency**	61.00 (57.00)	11.00 (10.28)	2.64 ± 1.47	1.25 ± 0.79	<0.001

^a^ Scores were obtained from the PFDI-20 questionnaire (questions number: 2, 3, 4, 7, 8, 15, 16, 17); ^b^ statistical significance.

**Table 3:** Preoperative and postoperative POP-Q findings (N=107).

Stage	Preoperative (%)	Postoperative (%)	P-value
**Anterior Compartments**			
**0**	0	0	<0.001
**1**	5 (4.67)	92 (85.98)	
**2**	60 (56.07)	14 (13.08)	
**3**	42 (39.25)	1 (0.93)	
**4**	0	0	
**Posterior Compartments**			
**0**	0	64 (59.81)	<0.001
**1**	13 (12.14)	38 (35.51)	
**2**	74 (69.15)	5 (4.67)	
**3**	20 (18.69)	0	
**4**	0	0	
**Apical Compartments**			
**0**	2 (1.86)	8 (7.47)	<0.001
**1**	53 (49.53)	97 (90.65)	
**2**	30 (28.03)	2 (1.86)	
**3**	19 (17.75)	0	
**4**	3 (2.80)	0	

**Table 4:** Frequency of procedures (N=107).

Procedure	Frequency (%)
**Rectocele repair**	107 (100)
**(Rectocele + Enterocele) repair**	70 (65.42)
**Cystocele repair**	70 (65.42)
**TVH ^a^**	21(19.62)
**Sacrospinous suspension**	46 (42.99)
**TVH + Sacrospinous suspension**	11 (10.28)
**High uterosacral suspension**	6 (5.60)
**TVH + High uterosacral suspension**	4 (3.73)
**TOT ^b^**	32 (29.90)
**TVT ^c^**	6 (5.60)
**(Cystocele + Enterocele + Rectocele) repair**	44 (41.12)
**TVH + (Cystocele + Enterocele + Rectocele) repair**	9 (8.41)

^a^ TVH: Transvaginal hysterectomy; ^b^ TOT: Transobturator tap; ^c^ TVT: Tension–free vaginal tap.

All subjects of this study underwent posterior vaginal compartment surgery (rectocele and/or enterocele repair) alone or simultaneously with surgery of other vaginal compartments.

During the 12-month follow-up, PFDI-20 scores before and after surgery were 141.87 ± 34.48 and 100.87 ± 26.48, respectively, which show the significant improvement of patients’ symptoms after surgery (p<0.001).

As shown in Table 2, all patients’ symptoms improved significantly after surgical treatment. For 37 (34.57%) patients, no cystocele repair was required, and only a posterior compartment repair with or without apical compartment repair was performed. In these patients, the PFDI-20 score indicates significant improvement at 12 months after surgery (PFDI-20 score before and after surgery: 131.70±36.51 and 98.22±20.54, respectively, p<0.001).

In this group of patients, symptoms such as obstructed defecation, urinary frequency, urinary urge incontinence and bulging showed significant improvement after surgery. Before and after surgery scores obtained based on the PFDI-20 questionnaire for obstructed defecation, urinary frequency, urinary urge incontinence and bulging are: 2.57 ± 1.50 and 1.54 ± 0.96 (p = 0.003), 1.92 ± 1.38 and 1.22 ± 0.58 (p = 0.007), and 2.92 ± 1.40 and 1.59 ± 1.01 (p < 0.001), 2.41 ± 1.49 and 1.08 ± 0.49 (p < 0.001), respectively.

During the vaginal examination based on the POP-Q system, as shown in Table 3, only one of the patients who underwent simultaneous surgery for anterior and posterior vaginal compartments repair and TVH as well as sacrospinous suspension showed a higher stage (stage 3) after surgery. For this patient, the following scores before surgery based on the POP-Q system were obtained in the examination performed after surgery: c = + 3, Ba = + 5, Bp = + 1, whereas D = + 1, Ba = + 2, Bp = -1.

Comparing the POP-Q results before and after surgery, significant improvements were seen at the 12-month follow-up (Table 3).

Surgery for SUI (TOT or TVT) was performed for 38 (35.51%) patients who were diagnosed by physical examination and urodynamic studies and provided informed consent for concomitant SUI surgery (Table 4). Seven of these patients did not complain of SUI in our history taking. However, their cough test in the clinical examination after prolapse replacement was positive, and SUI was shown at their urodynamic study. Thus, these patients underwent simultaneous surgery for SUI. After surgery, SUI was completely healed in all 7 patients.

In general, we observed significant differences between preoperative and postoperative symptoms of patients undergoing SUI surgery with surgery of other vaginal compartments at the same time (according to the scores regarding the SUI question in the PFDI-20 questionnaire, the preoperative and postoperative scores were 3.42 ± 1.77 and 1.24 ± 0.82, respectively; P<0.001). Three patients with SUI reported moderate urinary symptoms before the operation (based on the PFDI-20 questionnaire), but they showed no improvement after surgery, and their moderate symptoms were persisted.

The overall PFDI-20 score before surgery and 12 months after surgery showed significant improvements in the postoperative analysis of the posterior compartment surgery with each of the other vaginal compartment surgery, separately (p<0.001).

In the follow-up of the 107 patients under study, none of them showed any postoperative complications, so no other operation was performed.

## Discussion

This study was performed to investigate the outcomes of common vaginal route technique for posterior vaginal wall prolapse repair in the first Iranian Fellowship Teaching Center for female PFD. This technique is performed by attachment of the rectovaginal fascia to the pericervical ring using vaginal native tissue.

There are several techniques for repairing prolapses in the posterior vaginal compartment, yet there is no general agreement on the best surgical procedure. On the other hand, due to concerns about the side effects of using mesh when repairing this area, none of the surgical mesh products for POP vaginal repair have been approved by the Food and Drug Administration (FDA) for use in the United States [12]. So, finding the best surgical technique using native vaginal tissue seems necessary.

In a review article about the safety and efficiency of posterior vaginal wall surgeries, transvaginal midline fascial plication without levatorplasty has been suggested as the procedure of choice for posterior vaginal compartments repair. In this review, the authors found no support for using polypropylene mesh or biological graft. This study also reported that the full thickness attachment of the highest portion of the posterior vaginal wall (DeLancey level 3) to the uterosacral ligaments in patients with high rectoceles or rectoceles with posterior enterocele provides significant support [4].

In the Cochrane review article on “Surgery for women with posterior compartment prolapse”, the transvaginal repair is considered to be a more effective method in preventing recurrence of prolapses in the posterior vaginal wall after surgical treatments compared to the transanal repair, both objectively and subjectively. Also, this study concluded that evidence does not support the use of any mesh or graft materials for posterior vaginal wall repairs [13].

As shown in the results section of our study, according to both scores of the PFDI-20 questionnaire (subjective results) and clinical examinations (objective results), patients improved significantly at the 12-month follow-up after surgery using the common technique at our center.

Symptoms of posterior compartment prolapse, including bulging, obstructed defecation, splinting, and incomplete bowel emptying, also showed significant improvements at the 12-month follow-up after reconstructive surgery.

In our study, 79 (73.83%) patients diagnosed with POP who complained about urge urinary incontinence also showed significant improvements after POP reconstructive surgery.

Outcomes of a novel surgery technique developed for posterior vaginal compartment repair were examined in another study using a transvaginal technique with plication of the anterior rectal wall by suturing the rectal muscularis layer in a zig-zag pattern caudally. Symptoms of a prolapsed posterior vaginal compartment, including bulging, obstructed defecation, and wet overactive bladder (OAB) showed significant improvement at the 27±15-month follow-up after surgery (before surgery: 52.5%, 35.5%, and 21.6%; after surgery: 8.1%, 13.8%, and 10.6% for the above-mentioned symptoms, respectively). Similar to our results, significant anatomical improvements for posterior vaginal compartment prolapse were reported in this study [14].

Another study, a prospective cohort, investigated the effect of POP surgery on obstructed defecation symptoms at a 12-week follow-up. Splinting, straining, and incomplete emptying during defecating had improved significantly based on the PFDI-20 scores and POP-Q results [15].

In a study that focused on the main anatomical defects in the posterior vaginal compartment prolapses, the most common defects were associated with the vaginal vault (DeLancey level 1) and vaginal introitus (DeLancey level 3). These results are contrary to what has traditionally been emphasized so far, which related defects of the midvagina. So far, surgical outcomes of traditional posterior repair are more than needed based on DeLancey level 2 interventions, and less attention has been paid to DeLancey level 1 and 3 surgical interventions [16].

As mentioned before, the focus of the reconstructive method in our study center is on DeLancey level 1 and 3 repairs, which may have an impact on significant improvements shown anatomically and in PFDI-20 scores.

Based on data provided in another review article, rectocele reconstructive surgery should be performed using the native tissue transvaginal repair method to improve the anatomy and symptoms. In general, this study suggests that surgical interventions are needed for women with rectocele and obstructed defecation symptoms, and traditional native tissue posterior colporrhaphy through the vagina should be considered as the first option. In this study, the surgical technique commonly used in our study center was not particularly addressed [17].

In another study investigating in a retrospective manner the 3-month follow-up of patients who underwent site-specific colporrhaphy for posterior compartment prolapse repair, most defects were seen in the apical detachment of the rectovaginal fascia. This type of defect has been repaired by attachment of the rectovaginal fascia to the uterosacral ligaments (located outside the peritoneum) or the vaginal cuff. There were significant improvements at the short-term follow-up examinations, both anatomically and in terms of prolapse symptoms. However, in this study, significant improvement in urinary incontinence was observed after site-specific repairs in the apical and middle portions of patients who underwent posterior vaginal wall compartment surgery with or without repairs of prolapse of other vaginal compartments. However, no significant improvements were seen after the repair of inferior defects [18].

Our study may have several advantages; one of them is that all surgeries were performed using the same technique by a female PFD fellow assistant. Secondly, given the resources available to the authors of this study, this is the first time that the outcomes of this specific surgical technique have been examined at a 12-month follow-up. Finally, in this study about objective and subjective outcomes of patients, before and 12-month follow-up results were analyzed simultaneously.

Nevertheless, there were limitations to our study. One of these limitations is the short term follow-up. However, the authors will cover the long-term follow-up of patients understudy in the coming years. The other limitation is that only 107 patients out of the 180 patients completed their 12-month follow-up despite continuously reminding patients about coming to the clinic for the long-term follow-up in the initial follow-up sessions.

## Conclusion

Based on the results of the present study, it seems that the surgical procedure of rectovaginal fascia attachment to the pericervical ring in posterior vaginal wall prolapse repair can be used as an effective surgical intervention without significant morbidity.

Indeed, performing clinical trials and comparing this procedure with other surgical procedures in repairing posterior vaginal wall prolapse as well as long-term follow-up seems necessary in this regard. In our future study, we will report the outcomes of this surgical procedure after the long-term follow-up of these patients.

## Conflict of Interest

The authors declare that there is no conflict of interest.
